# Muscle metabolic remodeling in response to endurance exercise in salmonids

**DOI:** 10.3389/fphys.2014.00452

**Published:** 2014-11-21

**Authors:** Andrea J. Morash, Mark Vanderveken, Grant B. McClelland

**Affiliations:** ^1^Department of Biology, McMaster UniversityHamilton, ON, Canada; ^2^Institute for Marine and Antarctic Studies, University of TasmaniaHobart, TAS, Australia

**Keywords:** endurance exercise, salmonids, muscle remodeling, metabolism, migration, fuel selection

## Abstract

Phenotypic plasticity of skeletal muscle is relevant to swimming performance and metabolism in fishes, especially those that undergo extreme locomotory feats, such as seasonal migration. However, the influence of endurance exercise and the molecular mechanisms coordinating this remodeling are not well understood. The present study examines muscle metabolic remodeling associated with endurance exercise in fed rainbow trout as compared to migrating salmon. Trout were swum for 4 weeks at 1.5 BL/s, a speed similar to that of migrating salmon and red and white muscles were sampled after each week. We quantified changes in key enzymes in aerobic and carbohydrate metabolism [citrate synthase (CS), β-hydroxyacyl-CoA dehydrogenase (HOAD), hexokinase (HK)] and changes in mRNA expression of major regulators of metabolic phenotype (AMPK, PPARs) and lipid (carnitine palmitoyltransferase, CPT I), protein (aspartate aminotransferase, AST) and carbohydrate (HK) oxidation pathways. After 1 week of swimming substantial increases were seen in AMPK and PPARα mRNA expression and of their downstream target genes, CPTI and HK in red muscle. However, significant changes in CS and HK activity occurred only after 4 weeks. In contrast, there were few changes in mRNA expression and enzyme activities in white muscle over the 4-weeks. Red muscle results mimic those found in migrating salmon suggesting a strong influence of exercise on red muscle phenotype. In white muscle, only changes in AMPK and PPAR expression were similar to that seen with migrating salmon. However, in contrast to exercise alone, in natural migration HK decreased while AST increased suggesting that white muscle plays a role in supplying fuel and intermediates possibly through tissue breakdown during prolonged fasting. Dissecting individual and potentially synergistic effects of multiple stressors will enable us to determine major drivers of the metabolic phenotype and their impacts on whole animal performance.

## Introduction

Plasticity of skeletal muscle is integral to the physiological response of fishes to exercise. With chronic contraction there are a host of transcriptional changes leading to phenotypic plasticity of the muscle fibers that help ensure adequate oxygen and substrate delivery for ATP production to fuel the dynamic metabolism of this tissue (For review see McClelland, 2004). How muscle metabolism responds to exercise depends upon recruitment of different fiber-types (red vs. white), which is a function of the type and duration of the exercise (sprint vs. endurance; acute vs. chronic). Sub-maximal or low-intensity chronic exercise stimulates an increase in aerobic capacity (Johnston and Moon, 1980; Farrell et al., 1990) most likely through a number of molecular events involving a series of nuclear transcription factors such as nuclear respiratory factor (NRF) 1 and 2 and peroxisome proliferators-activated receptors (PPAR)-α, −β/δ, −γ and PPAR-γ cofactor (PGC)-1α that are implicated in mitochondria biogenesis and metabolic remodeling in mammalian muscle (Price et al., 2000; Baar et al., 2002). Data supporting a role for some of these factors in muscle remodeling from swim trained fishes are few and still equivocal (McClelland et al., 2006; LeMoine et al., 2010).

Submaximal chronic swimming is predicted to induce expression of key transcription factors such as PPARs. PPARs are nuclear receptors that can be activated by either elevated cellular levels of monounsaturated or polyunsaturated fatty acid ligands that, when bound, induce the PPARα-mediated expression of genes involved in the metabolism of lipids (Price et al., 2000).

Another main regulator of muscle energetic homeostasis is AMP-activated protein kinase (AMPK). AMPK works concurrently to stimulate fatty acid oxidation and glucose uptake in liver and muscle while inhibiting lipogenesis (Winder and Hardie, 1999). As a regulator of cellular energy production AMPK interacts with many of the regulatory centers of the major metabolic pathways. For example, AMPK stimulation increases PPARα and PGC-1α expression, citrate synthase activity (TCA cycle), hexokinase expression (glycolysis), carnitine palmitoyltransferase (CPT) I and β-hydroxyacl-CoA dehydrogenase (HOAD) (fatty acid oxidation) and cytochrome *c* (electron transport chain). Taken together, AMPK acts to stimulate ATP generating processes, particularly during exercise and fasting when an up-regulation of fat and carbohydrate oxidation is warranted.

Protein oxidation plays a relatively small role in ATP production until faced with extreme conditions. In migrating salmon, for example, protein oxidation (from muscle breakdown) begins to account for a growing proportion of the fish's metabolic fuel as lipid stores are depleted (Duncan and Tarr, 1958; Idler and Bitners, 1958; French et al., 1983). Aspartate transaminase (AST), which produces oxaloacetate for the TCA cycle (Hochachka and Somero, 1984), can be used as an indicator of protein oxidation capacity. Protein oxidation in satiated acutely exercised fish does not increase with swimming duration and contributes little to energy production (Alsop and Wood, 1997).

Muscle phenotype varies naturally in many fish species due to many factors including exercise. Probably the most extreme example of endurance exercise is that of the seasonal migration in Pacific salmon (10–2000 km), which requires specific metabolic alterations to ensure arrival at spawning grounds. However, the stressors encountered with spawning migrations are multifactorial including salinity changes, sexual maturation, and the cessation of feeding along side of chronic exercise. Current work on muscle metabolism in these animals indicates that there are specific temporal changes in transcription factors and key regulators of lipid, protein and carbohydrate oxidation (Morash et al., 2013), but it is unclear how exercise interacts with other influences such as fasting to influence this pattern. Recently we have uncovered some responses of muscle to long-term fasting in the closely related rainbow trout (Morash and McClelland, 2011). In the current study, we investigate long term exercise in fed rainbow trout to determine its effect(s) on the phenotypic plasticity red and white muscle.

Using rainbow trout (*Oncorhynchus mykiss*) as our model we examined the metabolic changes that take place during long-term exercise in fed fish. Specifically this research will determine spatial and temporal changes in transcription and activities of key metabolic enzymes central to energy metabolism in rainbow trout. We will relate these changes to those seen in trout with fasting Morash and McClelland (2011) with the goal of understanding their relative roles to changes seen in muscle with migration in a related salmonid, the Pacific salmon (Morash et al., 2013).

## Materials and methods

### Experimental species and exercise regime

All procedures were approved by the McMaster University Animal Research Ethics Board. Rainbow trout were obtained from a local trout hatchery (Humber Springs, Orangeville, ON), kept in 500 L tanks with circulating dechloriniated Hamilton tap water at 12°C and fed a commercial diet (Profishent Classic Floating Trout Grower, Martin Mills, Elmira, ON) twice daily to satiation. The exercised groups were introduced to the swim tunnel 1 week to acclimate prior to the beginning of exercise while control fish were maintained under routine conditions for at least 1 week prior to sacrifice. Fish were then exercised for 1, 2, or 4 weeks at 1.5 BL s^−1^ (approximately 50% U_crit_) for 23.5 h per day. Water flow was stopped twice for 15 min when the exercised trout were fed to satiation the same commercial trout feed as controls. Control fish were sampled after 1 week in routine conditions while swum fish were sampled after 1, 2, and 4 weeks of swimming. Both groups were euthanized by a blow to the head and then severing of the spinal cord. Length and weight were recorded. Condition factor (CF) was calculated using the following equation,

(1) $$\text{CF}=100w/l^{3}$$

where *w* is the weight of the fish in grams and *l* is the length of the fish in centimeters. Red and white muscle samples were excised and immediately frozen in liquid nitrogen and stored at −80°C until analysis.

### RNA extraction and cDNA synthesis

Frozen tissues were powdered in a liquid N_2_-chilled mortar and pestle. Total RNA was extracted from each tissue using TRIzol Reagent (Invitrogen, Carlsbad, CA) based on the acid guanidinium thiocyanate-phenol-chloroform extraction method. RNA was quantified by UV spectroscopy at 260 nm and then diluted to 0.5 μg/μl. cDNA was synthesized using 1 μg of DNase (Invitrogen, Carlsbad, CA) treated mRNA with SuperScript RNase H^−^ reverse transcriptase (Invitrogen, Carlsbad, CA) as described previously (Morash et al., 2008).

### Polymerase chain reaction (PCR) and sequencing

For each gene, available sequences from other fish species and mammals were aligned and PCR primers were designed using Primer3 software (Rozen and Skaletsky, 2000) within highly conserved regions. Each gene segment was amplified by PCR using 0.2 mM dNTP, 1.5 mM MgCl_2_, 0.5μM of each forward and reverse primer (Table 1), 1 unit of Taq polymerase (Fermentas, Burlington, Onatrio, Canada) and 1× Taq amplification buffer. All PCR products were verified through separation by gel electrophoresis using DNA size standards. Products were then purified using QiaQuick gel extraction kit (Qiagen, Mississauga, Ontario, Canada) and directly sequenced at the Mobix Lab (McMaster University) to ensure the proper sequence was being amplified.

**Table 1:** **Primer sequences used for real time PCR analysis**.

**Gene**	**5 ′ to 3 ′ Forward primer**	**5 ′ to 3 ′ Reverse primer**	**T_m_ °C**	**Size (bp)**
PPARα	ccaagttcagtttgccatga	attggggaagaggaaggtgt	60	173
PPARβ	ctggagctggatgacagtga	gtcagccatcttgttgagca	60	195
CPT Iβ 1	gatgttccgtgagggtagga	ttgtcttgcatggctctgac	58	80
CPT Iβ 2	gccgcaaactagagagagga	cccgtagtacagccacacct	58	199
CPT Iα1a	atgaggaatgccctcaagtg	gcttcctgccagagaacaac	58	120
CPT Iα1b	cgcttcaagaatggggtgat	caaccacctgctgtttctca	58	187
CPT Iα2	ccgttcctaacagaggtgct	acactccgtagccatcgtct	58	154
HK	ctgggacgctgaagaccaga	cggtgctgcatacctccttg	58	159
AST	gacctgtggctttgacttcc	gcaatctccttccactgctc	58	135
AMPK	actgtgttccgttggacagg	tcaatcatgagggcatcaaa	58	272
EF1α	cattgacaagagaaccattga	ccttcagcttgtccagcac	58	94

### mRNA quantification by real-time PCR

The expression of each mRNA was quantified using real time PCR with SYBR green plus ROX as a reference dye on a Stratagene Mx3000P (Stratagene, Texas, USA) real-time PCR system. Each 25μl reaction contained 12.5μL SYBR green mix, 1μL each of forward and reverse primers (5μM), 5.5μL of DNase/RNase free water and 5μL of 5× diluted cDNA (a no template control was included to ensure there was no contamination). Real-time PCR primers were designed using Primer3 software (Rozen and Skaletsky, 2000; Table 1). The thermal program included 3 min at 95°C, 40 cycles of 95°C for 15 s, 58°C for 30 s and 72°C for 30 s. A dissociation curve was performed to ensure only one PCR product was being amplified. Standard curves were constructed for each gene using serial dilutions of stock cDNA to account for any differences in amplification efficiencies. All samples were normalized to the housekeeping gene, EF1-α whose expression did not change significantly between time points.

### Enzyme analysis

All assays were performed in triplicate at room temperature in 96-well format using a Spectramax Plus 384 spectrophotometer (Molecular Devices, Sunnyvale, CA), and data was collected using Softmax Pro 4.7.1 software (Molecular Devices, Sunnyvale, CA). Frozen tissues were powdered using a liquid N_2_ chilled mortar and pestle and homogenized in 20 volumes of ice-cold homogenization buffer (100 mM potassium phosphate, 5 mM EDTA and 0.1% Triton at pH 7.2) using a glass on glass homogenizer chilled on ice. Homogenized samples were kept on ice prior to enzymatic analysis.

#### Citrate Synthase (CS)

CS was measured according to previously published protocols (McClelland et al., 2005). Briefly, the CS assay buffer contained (in mM) 20 TRIS (pH 8.0), 0.1 DTNB and 0.3 acetyl-CoA. The reaction was initiated by the addition of 0.5 mM oxaloacetate and absorbance was measured for 5 min at 412 nm. Control samples were assayed without oxaloacetate to control for background hydrolase activity.

#### β-hydroxyacyl-CoA Dehydrogenase (HOAD)

HOAD was measured according to previously published methods (McClelland et al., 2005) and consisted of (in mM) 50 imidazole (pH7.4), 0.1 acetoacetyl-CoA, 0.15 NADH and 0.1% Triton X-100 at 340 nm.

#### Hexokinase (HK)

The HK assay was modified from Houle-Leroy et al. (2000) for use in fish. The assay buffer contained (in mM) 4 ATP, 10 MgCl_2_, 0.5 NADP, 1 U glucose-6-phosphate dehydrogenase, in 50 HEPES (pH 7.0). The reaction was initiated by the addition of 5 mM D-glucose (omitted in control reactions).

### Statistical analysis

All statistical analyses were performed using SigmaStat v3.5 and Sigmaplot v12.5 (Systat Software Inc., San Jose, CA). All data were tested for normality and equal variance prior to performing One-Way ANOVA and Holm-Sidak post tests to test for significance between tissues and treatments. Significance level was set at *p* < 0.05.

## Results

### Condition of trout during experimental trials

Control trout showed no significant change in length, body weight or condition factor (CF) over the course of 4 week experimental period (Table 2). The exercised groups all showed a significant increase in their CF after their respective exercise regime. After 1 week of training, there was no significant change in length or weight of the fish, however, in combination the small changes lead to a significant increase in CF from 1.06 to 1.27. Two weeks of exercise significantly increased the mass of the fish but not the length leading to a CF o 1.25. By 4 weeks, exercise led to increases in both length and weight by 10 and 63%, respectively (Table 2; *p* < 0.05).

**Table 2:** **Physical characteristics of control trout and after 1, 2, or 4 weeks of exercise**.

	**Initial**	**Final**
**CONTROL**		
Length (cm)	16.10, 0.20	16.17, 0.39
Weight (g)	43.95, 2.09	47.75, 4.54
CF	1.05, 0.02	1.11, 0.05
**1 WEEK EXERCISE**		
Length (cm)	13.79, 0.39	14.12, 0.67
Weight (g)	28.54, 0.79	30.68, 5.15
CF	1.06, 0.06	1.27, 0.08*
**2 WEEKS EXERCISE**		
Length (cm)	15.16, 0.08	15.50, 0.39
Weight (g)	34.20, 1.03	45.13, 3.24*
CF	1.03, 0.04	1.21, 0.04*
**4 WEEKS EXERCISE**		
Length (cm)	16.50, 0.35	18.12, 0.38*
Weight (g)	46.17, 2.64	75.15, 5.08*
CF	1.02, 0.03	1.25, 0.04*

*Values are means ± SE. n = 5. Asterisks indicate significance between initial and final values. CF, condition factor*.

### Gene expression

**Figure 1:**
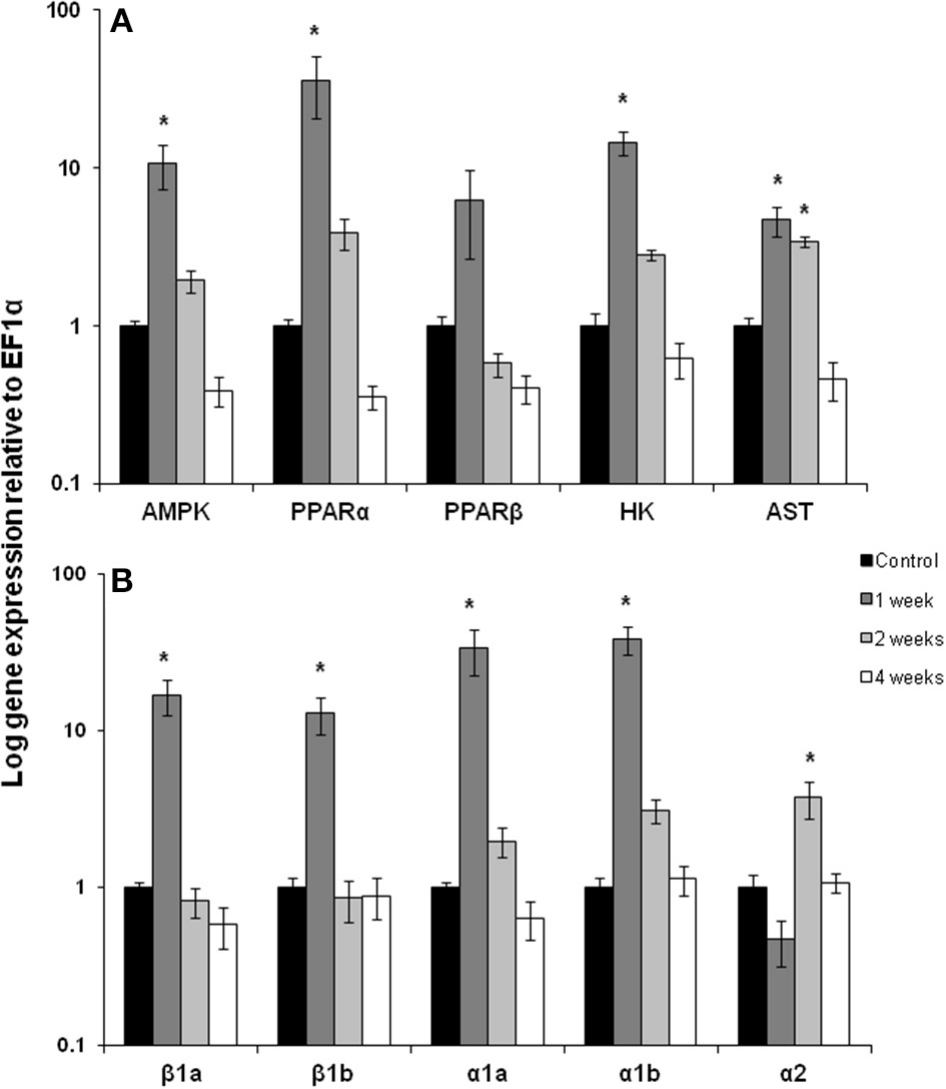
**Red muscle mRNA expression of (A) AMPK, AMP kinase; PPARα and β, peroxisome proliferator-activated receptor; HK, hexokinase; AST, aspartate amino transferase**. **(B)** carnitine palmitoyl-carnitine (CPT)I β 1a, β 1b, α1a, α1b, α2 in control, 1, 2, and 4 week exercised rainbow trout. Values are means ± *SE*. *n* = 4. Asterisk indicates significance between time points for each individual isoform.

After 1 week of chronic exercise, trout showed large and significant induction in mRNA expression for AMPK (10-fold), PPARα (36-fold), HK (15-fold), AST (5-fold) and the CPT 1 isoforms β 1a, β 1b, α1a, α1b (13–38-fold) in red muscle (Figures 1A,B; *p* < 0.05). Expression of all of these genes returned to control levels by week 2 of training except AST remained elevated and CPT Iα2 where expression increased only at this time point (Figures 1A,B; *p* < 0.05). Interestingly, the transcription factor PPARβ showed no significant change in mRNA expression with exercise (Figure 1A).

**Figure 2:**
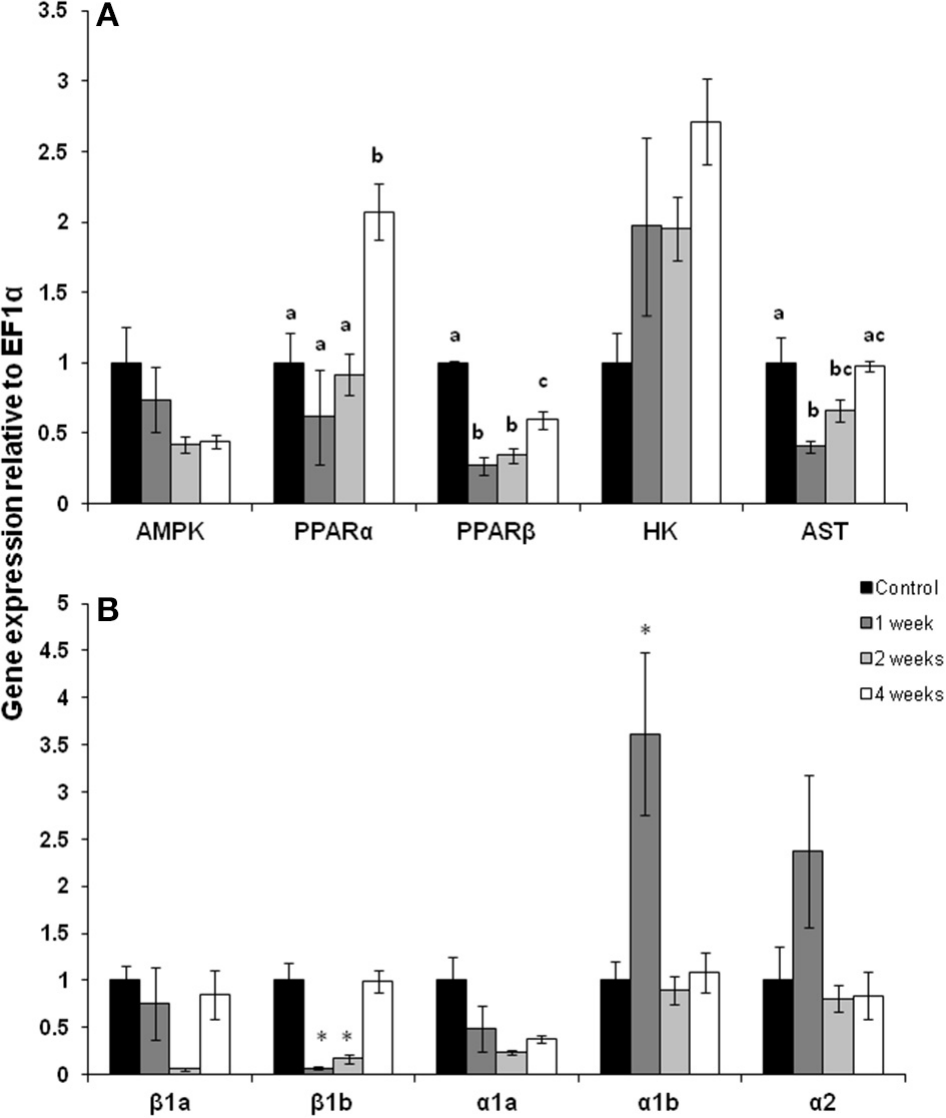
**White muscle mRNA expression of (A) metabolic pathway regulators and (B) CPT I isoforms in (A) AMPK, AMP kinase; PPARα and β, peroxisome proliferator-activated receptor; HK, hexokinase; AST, aspartate amino transferase**. **(B)** Carnitine palmitoyl-carnitine (CPT)I β 1a, β 1b, α1a, α1b, α2 in control, 1, 2, and 4 week exercised rainbow trout. Values are means ± *SE*. *n* = 4. Asterisks or letters indicate significance between time points for each individual isoform.

The pattern of gene expression was distinct in white muscle with PPARα mRNA expression doubling but only after 4 weeks of exercise (Figure 2A; *p* < 0.05). In contrast, PPARβ mRNA expression was 50% lower after 1, 2, and 4 weeks of exercise (Figure 2A; *p* < 0.05). There was a trend for AMPK expression to decrease throughout the training period but this decline was not statistically significant. AST mRNA expression fell by 50% after 1 week of exercise, remained low at 75% of control expression after 2 weeks but returned to control expression levels by 4 weeks of training (Figure 2A; *p* < 0.05). The expression of CPT I isoforms was variable with CPT Iβ 1b decreasing at weeks 1 and 2, while CPT Iα1b increased by 350% at week 1 Figure 2B; *p* < 0.05).

### Enzyme activity

**Figure 3:**
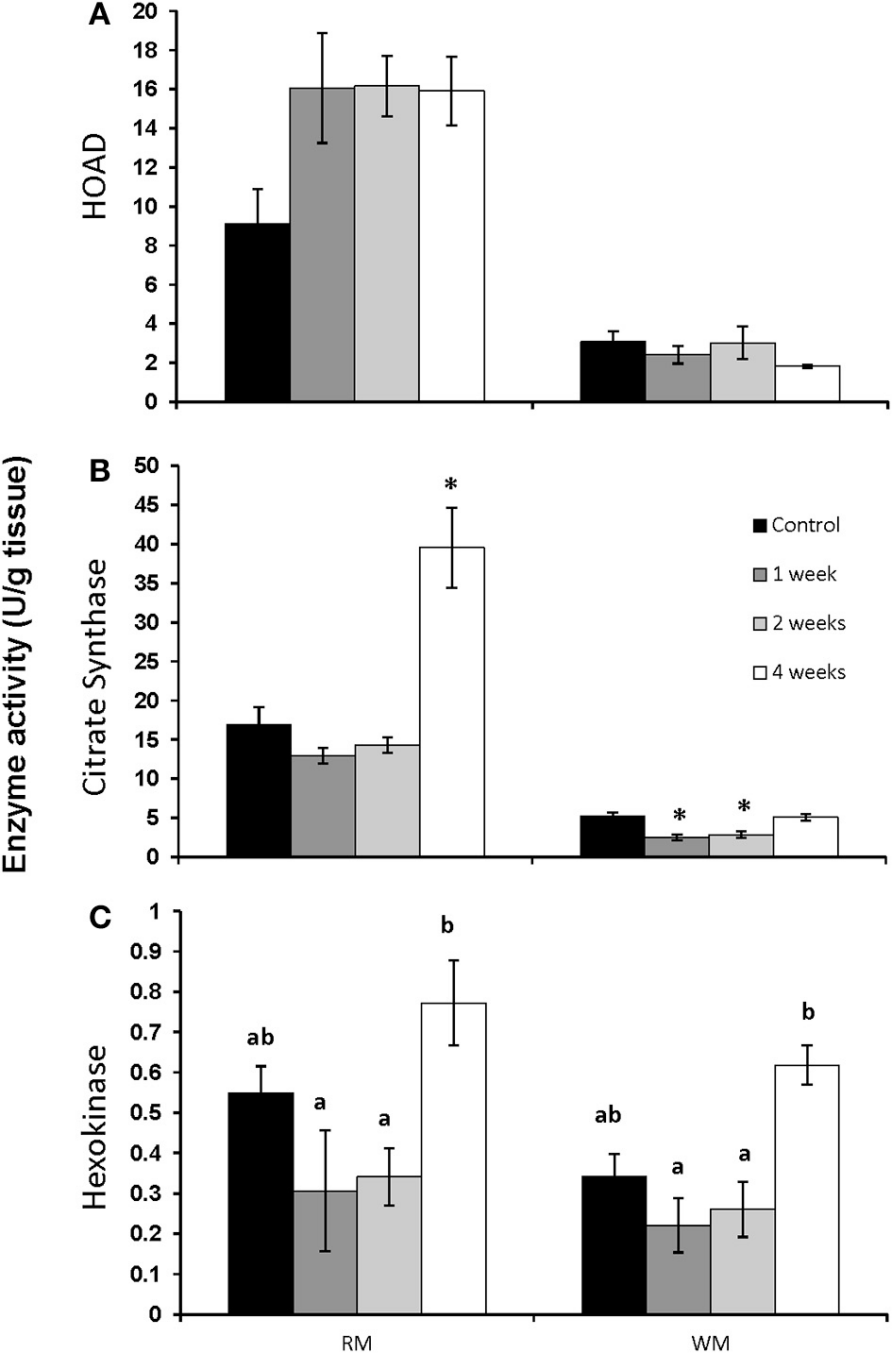
**Maximal enzyme activities (apparent Vmax) in red and white muscle from control, 1, 2, and 4 week exercised rainbow trout**. **(A)** β-hydroxyacyl-CoA dehydrogenase (HOAD), **(B)** citrate synthase and **(C)** hexokinase. Values are means ± *SE*. *n* = 4. Asterisks represent significantly different from controls, and bars that do not share a letter are significantly different from each other within each tissue.

The activity of HOAD in both red and white muscle was unaffected by chronic swimming (Figure 3A). CS activity did not change in red muscle until 4 weeks of training when it increased significantly from controls (Figure 3B; *p* < 0.05). In white muscle, CS was significantly lower after 1 and 2 weeks of exercise (Figure 3B; *p* < 0.05). HK activity significantly increased in both red and white muscle after 4 weeks of exercise when compared to 1 and 2 week exercised fish (Figure 3C; *p* < 0.05).

## Discussion

Chronic swimming in fed trout leads to a metabolic remodeling of skeletal muscle that varied both spatially between fiber-types and temporally over a 4 week period. Red muscle showed a very large (up to 10×) transcriptional response after only a week of exercise, but changes in maximal activities of enzymes occurred only after 4 weeks of exercise. In contrast, white muscle gene expression was generally reduced throughout the duration of training with the exception of an increase in PPARα at 4 weeks. Similar to transcription, there were either no changes or decreased enzymatic activity in white muscle, except for HK which increased only after 4 weeks. Exercise is an important stimulator of grown in fish and trout did show increased growth by 4 weeks of training. The duration and intensity of the exercise in this study closely mimics that of migrating salmon. However, the extent to which individual factors of migration, such as endurance exercise and fasting, affect muscle physiology and metabolism is currently unknown. Although exercise leads to muscle remodeling in trout, changes that occur with migration likely result from a combination of multiple stressors including muscle contraction and fasting.

During the first week of chronic swimming, red muscle mRNA expression was significantly elevated for many of the genes measured (Figure 1). In particular, two of the main regulators of lipid oxidation, AMPK and PPARα, (Figure 1A) increased, along with their downstream targets. PPARα is likely responsible (Price et al., 2000) for the increase in CPT I mRNA expression at the same time point, while AMPK plays a role in stimulating gene expression from both fatty acid (CPT I) and glycolytic pathways (HK) (Figures 1A,B). These modifications suggest that the red muscle is predominantly geared toward lipid oxidation to fuel muscle contraction, a common response to exercise training (Johnston and Moon, 1980; Farrell et al., 1990). At the same time, changes in white muscle gene expression were more variable, showing no significant changes due to swim training, except for CPT I α1b. Similarly, the expression and activity of important fatty acid oxidation genes and proteins were also highest in red muscle in migrating salmon during the first week of migration while they were still in the ocean traveling to the river mouth (Morash et al., 2013). Indeed this may be a common transcriptional response to muscle contraction in fishes. Zebrafish also show a stimulation of muscle transcription during the early stages of swim training (LeMoine et al., 2010). In contrast, long-term fasting in trout led to little change in the expression of PPARα or PPARβ in red and white muscle (Morash and McClelland, 2011). However, it is unclear if transcription is stimulated immediately upon cessation of feeding or how combined fasting and exercise interact at the level of skeletal muscle.

The up-regulation of metabolic regulators, AMPK and PPARα, corresponded to induction in the expression of their downstream targets and suggests that changes in their transcription with exercise, but the role of post-translational modifications of these regulators in unclear. The expression and downstream effects of AMPK and PPARs in fish are still not well characterized. However, recent research has found that increased AMPK activity in exercising trout muscle facilitates downstream target gene expression (Magnoni et al., 2014). Furthermore, whole genome duplication events have resulted in multiple isoforms of factors such as PPARs (and likely AMPK), many of which have yet to be characterized (Batista-Pinto et al., 2005; Leaver et al., 2005, 2007). Large scale transcriptomic analysis of muscles from resting and exercising fish, including migrating salmon, are emerging in the literature and will help to clarify our understanding of these molecular mechanisms (Miller et al., 2009; Palstra et al., 2013). However, salmon appear to express the same multiple paralogs of CPT I originally characterized in trout (Morash et al., 2010, 2013). CPT I is transcriptionally regulated by PPARα in mammals (Price et al., 2000), but appears to be more complex in fish as the expression of CPT I α1b isoform in white muscle was activated independently of PPARα expression. CPT I may respond independently to stresses such as exercise, migration or fasting.

The significant increase in AMPK, PPARα and the CPT I isoforms seen here in trout, suggests that the capacity for fatty acid oxidation would be increased in the red muscle of these well fed, exercising fish. The increase in an enzyme used as a marker of β-oxidation capacity (HOAD) showed an almost doubling in activity within the first week of exercise, although this increase did not reach statistical significance (Figure 3A). The effects of short-term chronic exercise may be influencing the mRNA expression and enzyme activity during the early stages of migration (Morash et al., 2013). During the initial phase of migration, salmon have large lipid stores and therefore the available lipid supply should be similar to fed trout (French et al., 1983). Moreover, the low intensity swimming during our exercise protocol and early in migration would be primarily powered by lipid and protein oxidation (Johnston and Moon, 1980; French et al., 1983; Lauff and Wood, 1996). The increased expression of AST mRNA expression in the first week of chronic exercise in red muscle (Figure 1A) may serve to support this protein oxidation. In contrast there was no increase in AST mRNA expression in white muscle likely due to a low contribution to low intensity chronic swimming (Rome et al., 1984). Similar changes in AST mRNA expression in both exercised trout and migrating salmon red muscle suggest that protein oxidation in these tissues may be most affected by exercise and not fasting (Morash et al., 2013). In contrast, AST mRNA expression was 10 times higher in the white muscle of migrating salmon during the first week, which is potentially a result of fasting during the migration or a reliance on periodic burst swimming.

The initial induction of transcription seen early on in swim training were largely absent by 2 and 4 weeks and in some cases even reduced below constitutive expression levels in white muscle of trout. Similar to other fishes (LeMoine et al., 2010) and mammals (Mathai et al., 2008; Perry et al., 2010) changes in transcription and protein expression are temporally separated. By 4 weeks of swim training CS activity more than doubled in red muscle (Figure 3) consistent with past studies on fishes showing that mitochondrial biogenesis can occur between 1 and 4 weeks of training (Farrell et al., 1990; McClelland et al., 2006; Palstra et al., 2010). Similarly, transcript levels were highest at early stages of migration with the exception of red muscle AMPK mRNA expression which increases later in migration, as does HK expression and activity (Morash et al., 2013). Furthermore, there is an increase in AST expression in white muscle of migrating salmon as they begin to catabolize and degrade muscle proteins after long-term swimming in the fasted state (Mommsen et al., 1980; French et al., 1983).

We have discussed the effects of chronic exercise on the phenotypic plasticity of skeletal muscle in rainbow trout and compared these to wild migrating Pacific salmon in an attempt to elucidate the individual effects of exercise on the metabolic alterations in the muscle that occur in these populations (Morash et al., 2013). While there are several similarities and differences in responses we are limited in our conclusions as migration is a multifactorial process that is influenced by changes in salinity, sexual maturation, fasting, and exercise. However, our data sheds some light on the individual effect of long term exercise in salmonids that may be contributing to the large scale metabolic changes in migrating salmon.

In conclusion, low intensity chronic exercise results in phenotypic plasticity of skeletal muscle, predominantly in red muscle with little effect on mRNA expression or enzyme activity of target enzymes in white muscle. While we found mRNA expression of transcription factors and their downstream targets to be induced during the first week of exercise while enzyme activity changes occurred later on to primarily increase muscle capacity for lipid oxidation and aerobic metabolism. Changes in markers of protein oxidation suggest that proteins may contribute to intermediate supply for the TCA cycle to fuel oxidative metabolism in the red muscle. There was a similar temporal transcriptional response in the red muscle of migrating salmon indicating that exercise may dominate the regulation of metabolism during early stages of migration. After 4 weeks of exercise there was an increase in mitochondrial biogenesis in the red muscle of trout as a result of exercise training. We did not find any changes in white muscle after 4 weeks of exercise unlike the white muscle during the final stages of migration in salmon where there is an increase in glycolytic enzyme and protein oxidation capacity. This is likely a result of long-term fasting and the depletion of lipid stores. Understanding the role of exercise in the remodeling of skeletal muscle has important implications for unraveling the complex physiological changes that occur in naturally migrating species but also for improving aquaculture practices to maximize muscle growth and fish health.

### Conflict of interest statement

The authors declare that the research was conducted in the absence of any commercial or financial relationships that could be construed as a potential conflict of interest.
